# 

**Published:** 1885-08

**Authors:** 


Whole Number, 308. AUGUST, 1885. Vol. LI., Number 2.

INDEX MEDICUS

### THE CHICAGO MEDICAL JOURNAL AND EXAMINER

LIBRARY INDEXED S.G.O.

EDITORS:

S. J. JONES, M.D., LL.D.

W. W. JAGGARD, A.M., M.D. HAROLD N. MOYER, M.D.

CHICAGO:

PUBLISHED MONTHLY BY THE CHICAGO MEDICAL PRESS ASSOCIATION.

240 WABASH AVENUE.

CLARK & LONGLEY, PRINTERS, 163 AND 165 DEARBORN STREET, CHICAGO.

